# Supplementary material for: Topological turning points across the human lifespan
Source: Nat Commun. 2025 Nov 25;16:10055. doi: 10.1038/s41467-025-65974-8 (PMC12647875; doi:10.1038/s41467-025-65974-8)
Supplement: Supplementary file 1 — Supplementary Information [file 41467_2025_65974_MOESM1_ESM.pdf]

Supplementary Information:

Topological turning points across the human lifespan

Alexa Mousley[1*], Richard A.I. Bethlehem[2], Fang-Cheng Yeh[3] & Duncan E. Astle[1,4]

[1]MRC Cognition and Brain Sciences Unit, University of Cambridge, Cambridge, UK
[2]Department of Psychology, University of Cambridge, Cambridge, UK
[3]Department of Neurological Surgery, University of Pittsburgh, Pittsburgh, PA, USA
[4]Department of Psychiatry, University of Cambridge, Cambridge, UK

*Corresponding author: Alexa Mousley
Email: alexa.mousley@mrc-cbu.cam.ac.uk
Address: 15 Chaucer Road, Cambridge, CB2 7EF UK

Supplementary Table 1. Individual dataset demographics, scanning details, and preprocessing method.

	Demographics				Imaging	
	**Total Scans**	**Analysis Sample**	**Ages**	**Sex**	**dMRI Parameters** (b-value: # directions)	**Preprocessing**
**Developing Human Connectome Project (dHCP)** (Edwards et al., 2022)	447	446	0 – 7w<br>*M* = 0.02<br>SD = 0.02	F = 46%<br>M = 54%	400 s/mm^2: 64<br>1000 s/mm^2: 88<br>2600 s/mm^2: 128<br>(Tournier et al., 2020)	https://brain.labsolver.org/hcp_d2.html
**Baby Connectome Project (BCP)** (Howell et al., 2019)	179	92	1 – 5y<br>*M* = 1.74<br>SD = 0.08	F = 45%<br>M = 55%	500 s/mm^2: 9<br>1000 s/mm^2: 12<br>1500 s/mm^2: 17<br>2000 s/mm^2: 24<br>2500 s/mm^2: 1234<br>3000 s/mm^2: 48<br>or<br>700 s/mm^2: 36<br>1500 s/mm^2: 48<br>3000 s/mm^2: 60	Processed in-house (QSIprep)
**Centre for Attention Learning and Memory (CALM)** (Holmes et al., 2019)	392	77	6 – 18y<br>*M* = 10.28<br>SD = 2.18	F = 66%<br>M = 34%	1000 s/mm^2: 64	Processed in-house (QSIprep)
**Resilience in Education and Development (RED)** (Johnson et al., 2022)	75	74	7 – 12y<br>*M* = 8.58<br>SD = 1.01	F = 58%<br>M = 42%	1000 s/mm^2: 68	Processed in-house (QSIprep)
**Attention and Cognition in Education (ACE)** (Bathelt et al., 2019)	85	84	7 – 13y<br>*M* = 10.04<br>SD = 1.58	F = 50%<br>M = 50%	1000 s/mm^2: 60	Processed in-house (QSIprep)
**Human Connectome Project Development (HCPd)** (Somerville et al., 2018)	633	633	6 – 22y<br>*M* = 14.50<br>SD = 4.06	F = 54%<br>M = 46%	1500 s/mm^2: 92, 93<br>3000 s/mm^2: 92, 93<br>(Harms et al., 2018)	https://brain.labsolver.org/hcp_d.html
**Human Connectome Project Young Adult (HCPya)** (Van Essen et al., 2013)	1065	1062	22 – 37y<br>*M* = 28.74<br>SD = 3.67	F = 54%<br>M = 46%	1000 s/mm^2: 90<br>2000 s/mm^2: 90<br>3000 s/mm^2: 90<br>(Harms et al., 2018)	https://brain.labsolver.org/hcp_ya.html
**Human Connectome Project Ageing (HCPa)** (Bookheimer et al., 2019)	706	705	36 – 90y<br>*M* = 59.76<br>SD = 14.92	F = 56%<br>M = 44%	1500 s/mm^2: 92, 93<br>3000 s/mm^2: 92, 93<br>(Harms et al., 2018)	https://brain.labsolver.org/hcp_a.html
**Cambridge Centre for Ageing and Neuroscience (CamCAN)** (Shafto et al., 2014)	634	629	19 – 89y<br>*M* = 55.34<br>SD = 18.55	F = 50%<br>M = 50%	1000 s/mm^2: 30<br>2000 s/mm^2: 30	https://brain.labsolver.org/camcan.html

Note: The analysis sample size indicates all scans retained after removing longitudinal scans (BCP), neurodivergent participants (CALM), and density outliers. For age, 'y' indicates years, 'w' indicates weeks, '*M*' is the mean age, and 'SD' is the standard deviation. For sex, 'F' indicates female, and 'M' indicates male.

Supplementary Table 2. Brief summary of all graph theory metrics used.

	Metric	Brief Description	BCT function
Global Measures	Density	Percent of connections present	density_und()
	Modularity	Subdivision of network into nonoverlapping nodes (Neman's Spectral Community Detection)	modularity_und()
	Global Efficiency	Average inverse shortest path length	charpath() – second output
	Characteristic Path Length	Average shortest path length	charpath() – first output
	Core/Periphery Structure	Subdivide network into two nonoverlapping groups: (1) dense core and (2) sparse periphery	core_periphery_dir()
	Small-Worldness	Ratio between clustering coefficient and characteristic path length, normalized with null models (values > 1 indicates presence of small-worldness)	custom function using randmio_und() for null models
	K-Core	Largest subnetwork comprising of nodes with degree *k* or larger ($k = 6$)	kcore_bu()
	S-Core	Largest subnetwork comprising of nodes with strength *s* or larger ($s = 0.6$)	score_wu()
Local Measures	Strength	Sum of edge weights	strengths_und()
	Local Efficiency	Global efficiency calculated in neighborhood of nodes	efficiency_wei()
	Clustering Coefficient	Fraction of neighbors that are neighbors with each other	clustering_coef_wu()
	Betweenness Centrality	Fraction of all shortest paths that pass through each node	betweenness_wei()
	Subgraph Centrality	Weighted sum of all close walks starting and ending at the node	subgraph_centrality()

Note: Brief descriptions are based on definitions from Rubinov and Sporns (2010).

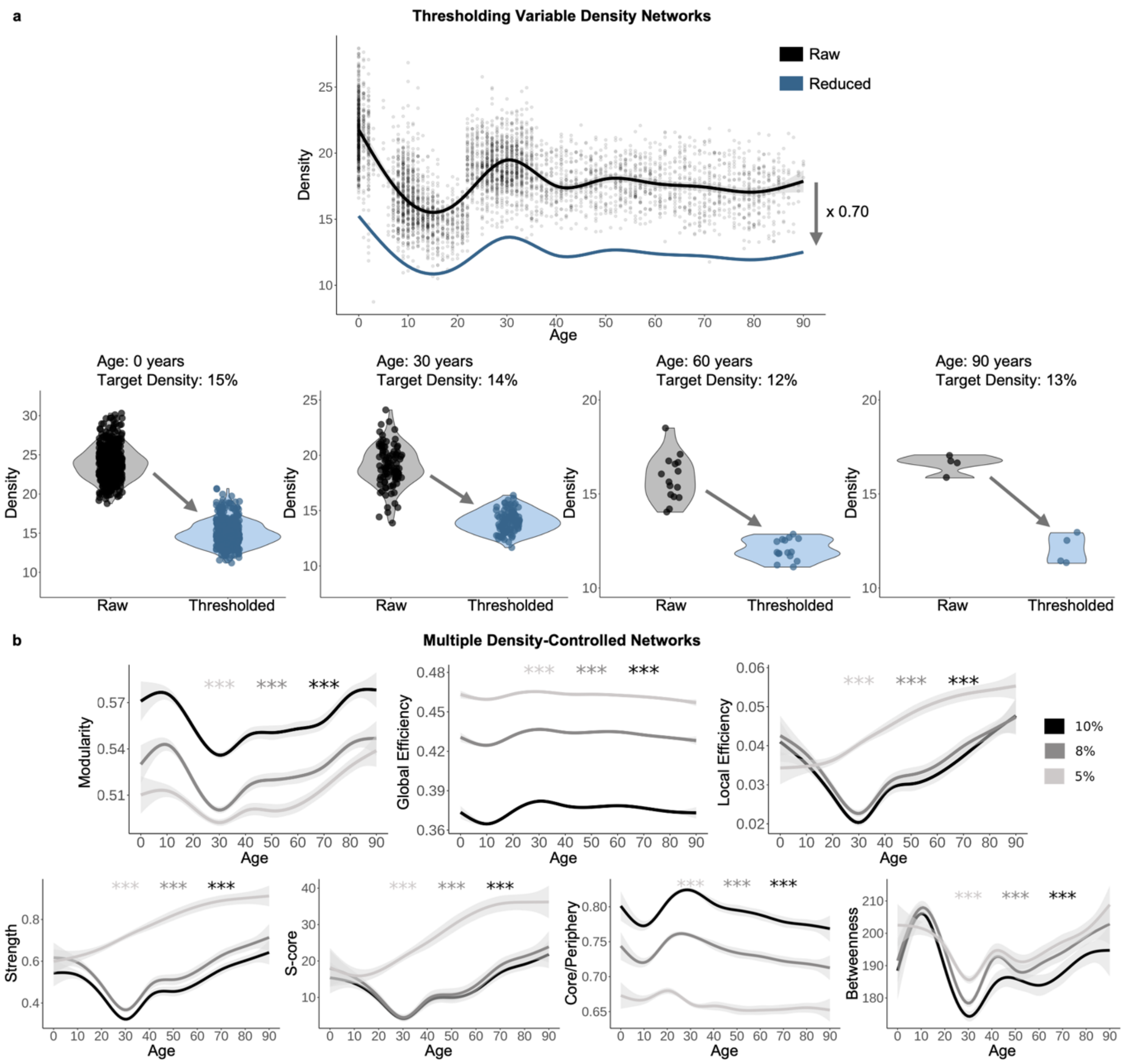

Supplementary Figure 1. Variable density thresholding procedure and exploration of multiple controlled-density levels. (a) Generalized additive model of unthresholded network density ('Raw') while controlling for sex, dataset, atlas, and neurodiversity group (neurotypical or neurodiverse). This regression was reduced by 30% (multiplied by 0.70), which was used to get age-specific target densities for thresholding ('Reduced'). Violin plots show examples of density for age bins 0, 30, 60, and 90 years before thresholding (black) and after thresholding to target density (blue). **(b)** Sensitivity analysis for density-controlled thresholding where all networks have the same *exact* density. Seven topological measures significantly change across age across controlled density to 10%, 8%, and 5%. Trajectories indicate 10% and 8% have similar patterns, whereas very sparse networks (5%) show different patterns on some but not all metrics. *** indicates $p < 0.001$.

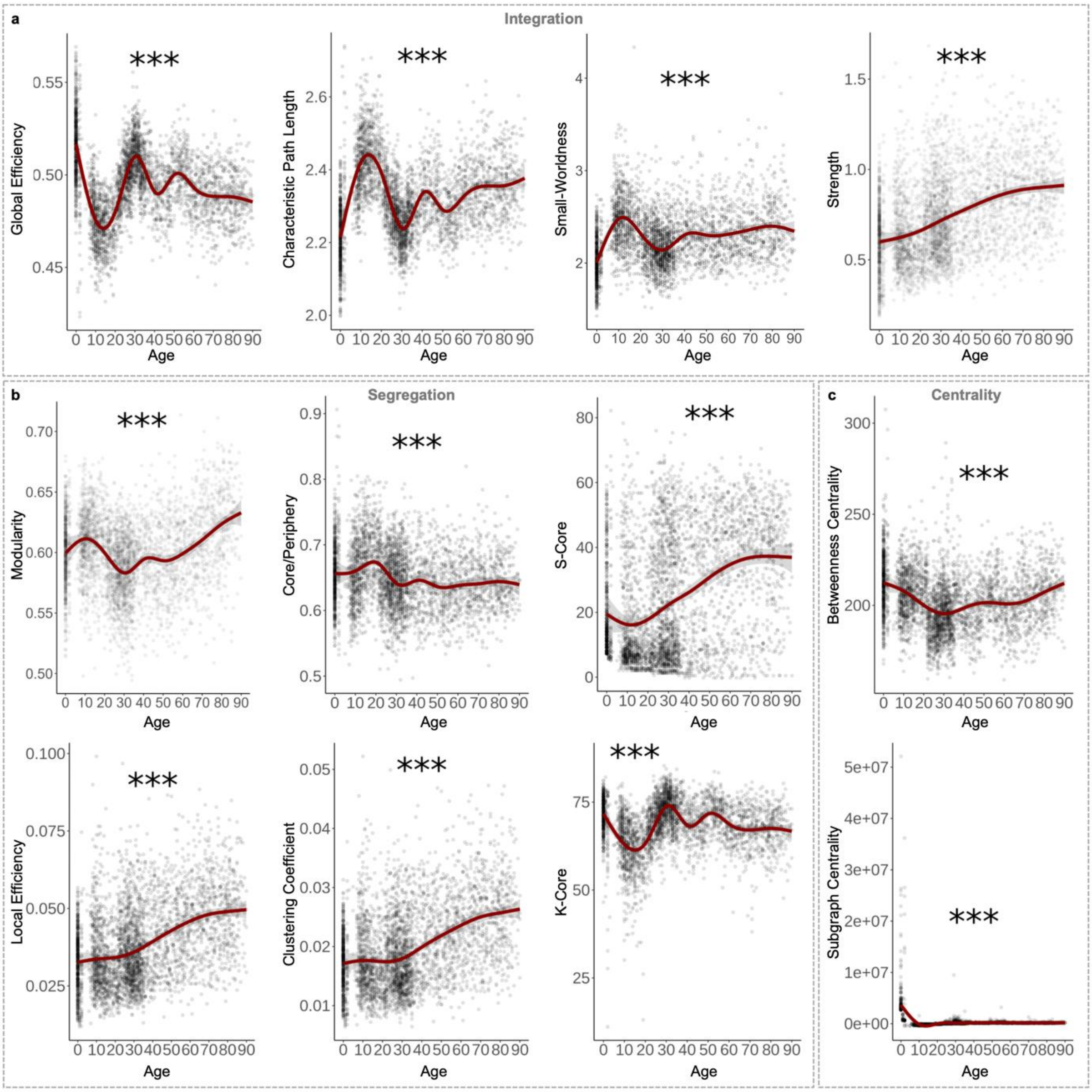

Supplementary Figure 2. Topological changes across the lifespan with variable density networks. (a) Global efficiency fluctuates across the lifespan, with high points at birth and 30 years old and declining in late life. Characteristic path length and small-worldness display the inverse pattern of global efficiency. The average strength of networks significantly increases across the lifespan. **(b)** Modularity hits a lifetime low around 30 years old before steadily increasing for the rest of the lifespan. Core/Periphery structure peaks around 20 years old. S-Core significantly increases across the lifespan. Average local efficiency and clustering coefficient significantly increase across the lifespan. K-Core varies across the lifespan with relative peaks around 30 and 50 years old. **(c)** Average betweenness centrality displays a lifetime low around 30 years old. Average subgraph centrality starts high at birth and significantly decreases across the lifespan. Shaded area around best fit lines represent 95% confidence intervals. *** indicates $p < 0.001$, ** indicates $p < 0.01$, * indicates $p < 0.05$.

Supplementary Table 3. Generalized additive models of graph theory metrics with variable density networks.

		F(metric,age)	Estimated df	*p-value*
**Integration**	Global efficiency	231.40	8.91	$p < 2.00 \times 10^{-16}$
	Characteristic path length	219.90	8.89	$p < 2.00 \times 10^{-16}$
	Small-Worldness	68.03	8.62	$p < 2.00 \times 10^{-16}$
	Average strength	33.10	5.46	$p < 2.00 \times 10^{-16}$
**Segregation**	Modularity	71.37	8.00	$p < 2.00 \times 10^{-16}$
	Core/Periphery	16.35	8.18	$p < 2.00 \times 10^{-16}$
	S-Core	38.77	5.36	$p < 2.00 \times 10^{-16}$
	Local efficiency	60.28	4.50	$p < 2.00 \times 10^{-16}$
	Clustering coefficient	67.44	5.18	$p < 2.00 \times 10^{-16}$
	K-Core	135.30	8.85	$p < 2.00 \times 10^{-16}$
**Centrality**	Betweenness centrality	37.73	6.66	$p < 2.00 \times 10^{-16}$
	Subgraph centrality	19.15	8.53	$p < 2.00 \times 10^{-16}$

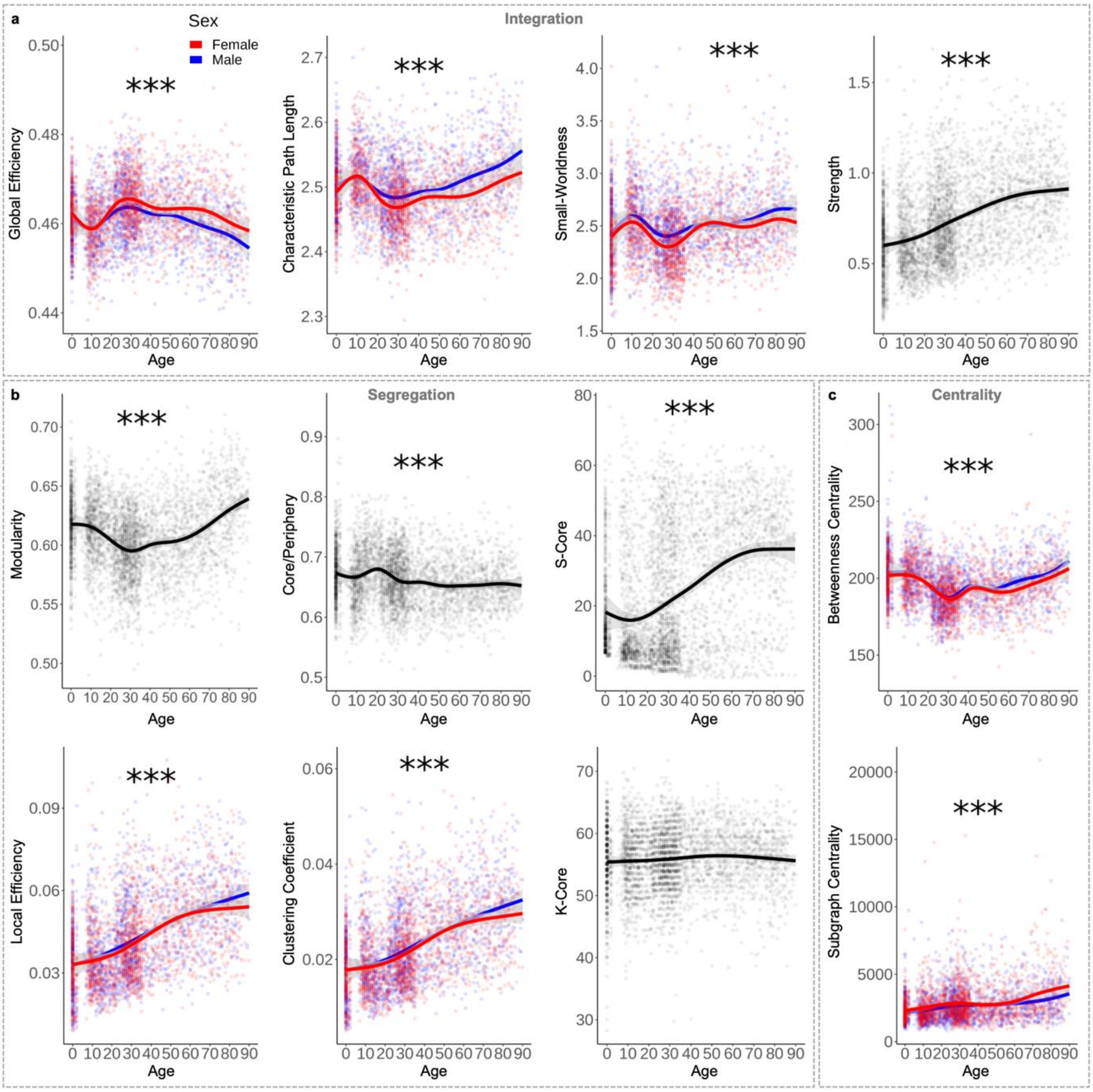

Supplementary Figure 3. Significant sex effects in controlled-density network topology. **(a)** Global efficiency (group estimate = 1.44 x 10^{-03}, t-value = 6.71, p = 2.18 x 10^{-11}), characteristic path length (group estimate = -0.06, t-value = -6.36, p = 2.27 x 10^{-10}), and small-worldness (group estimate = -0.06, t-value = -9.34, p < 2.00 x 10^{-16}) had significant sex effects across the lifespan with females showing higher efficiency, shorter path lengths and lower small-worldness than males from about 20 years old. Average network strength, however, had no significant effects of sex (p = 0.543). **(b)** Neither modularity (p = 0.761) nor core/periphery structure (p = 0.110) showed a significant effect of sex. S-core (p = 0.258) and K-core (p = 0.146) did not have significant effects of sex. Average local efficiency (group estimate = -1.05 x 10^{-03}, t-value = -2.42, p = 0.016) and clustering coefficient (group estimate = -6.37 x 10^{-04}, t-value = -2.68, p = 0.007) did have significant effects of sex, with females showing lower clustering and local efficiency compared to males particularly in late life (70 years old and older). **(c)** Both centrality metrics had significant effects of sex. Females had lower average betweenness centrality (group estimate = -1.12, t-value = -2.33, p = 0.020) compared to males after about 50 years old and significantly higher subgraph centrality after about 60 years of age (group estimate = 207.23, t-value = 4.99, p = 6.24 x 10^{-07}). Shaded area around best fit lines represent 95% confidence intervals. *** indicates p < 0.001, ** indicates p < 0.01, * indicates p < 0.05.

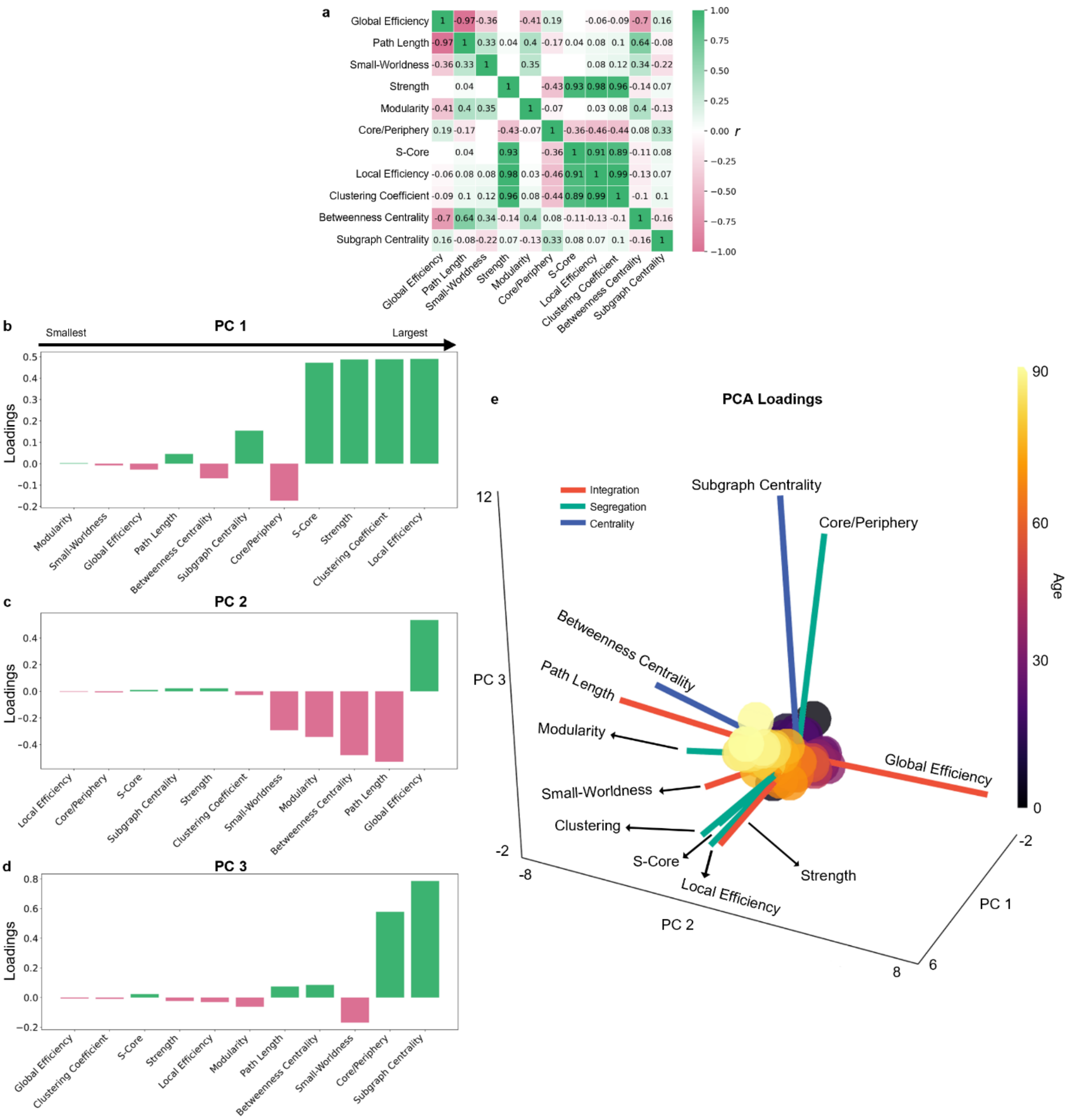

Supplementary Figure 4. Principal components analysis of network organization. (a) Graph theory measures convey unique and redundant network topology information, as significant Pearson correlations indicate. White boxes indicate no significant correlation. All loading scores are ordered from smallest to largest for **(b)** PC 1, **(c)** PC 2, and **(d)** PC 3. **(e)** Three-dimensional biplot showing PCA loadings with the age-averaged PCA scores.

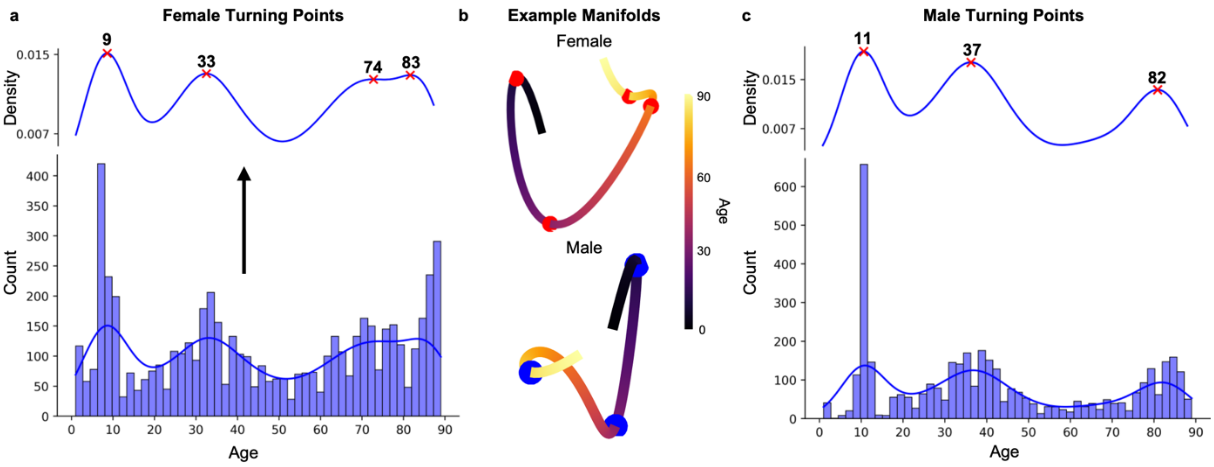

Supplementary Figure 5. Sex-stratified turning points. (a) Across all turning points identified in female manifolds, major turning points converge around nine, 33, 74, and 83 years old. **(b)** Lines of best fit through example female and male manifolds. Red spheres identify turning points for females and blue spheres for males. **(c)** Major turning points occurred around 11, 37, and 82 years old across all turning points for males.

Supplementary Table 4. ***p-values*** **for all correlations in each epoch.**

		Pearson correlation *p-values*				
		**Epoch 1**	**Epoch 2**	**Epoch 3**	**Epoch 4**	**Epoch 5**
**Integration**	Global Efficiency	2.65×10^{-13}	7.96×10^{-47}	2.40×10^{-05}	0.013	0.510
	Characteristic Path Length	4.02×10^{-11}	1.98×10^{-43}	2.05×10^{-06}	0.014	0.545
	Small-Worldness	2.26×10^{-76}	6.14×10^{-98}	3.54×10^{-09}	0.581	0.096
	Strength	6.93×10^{-04}	6.22×10^{-19}	7.79×10^{-16}	0.850	0.789
**Segregation**	Modularity	0.141	2.84×10^{-37}	4.09×10^{-06}	1.83×10^{-04}	0.551
	Core/Periphery	2.34×10^{-08}	0.015	0.003	0.469	0.933
	S-Core	0.676	7.39×10^{-12}	2.03×10^{-17}	0.796	0.974
	Local Efficiency	0.001	1.82×10^{-17}	1.26×10^{-20}	0.469	0.867
	Clustering Coefficient	0.006	3.71×10^{-15}	2.07×10^{-21}	0.276	0.832
**Centrality**	Betweenness Centrality	0.006	1.10×10^{-71}	1.85×10^{-04}	0.045	0.730
	Subgraph Centrality	0.320	6.57×10^{-09}	0.134	0.085	0.010

Note: All values are uncorrected.

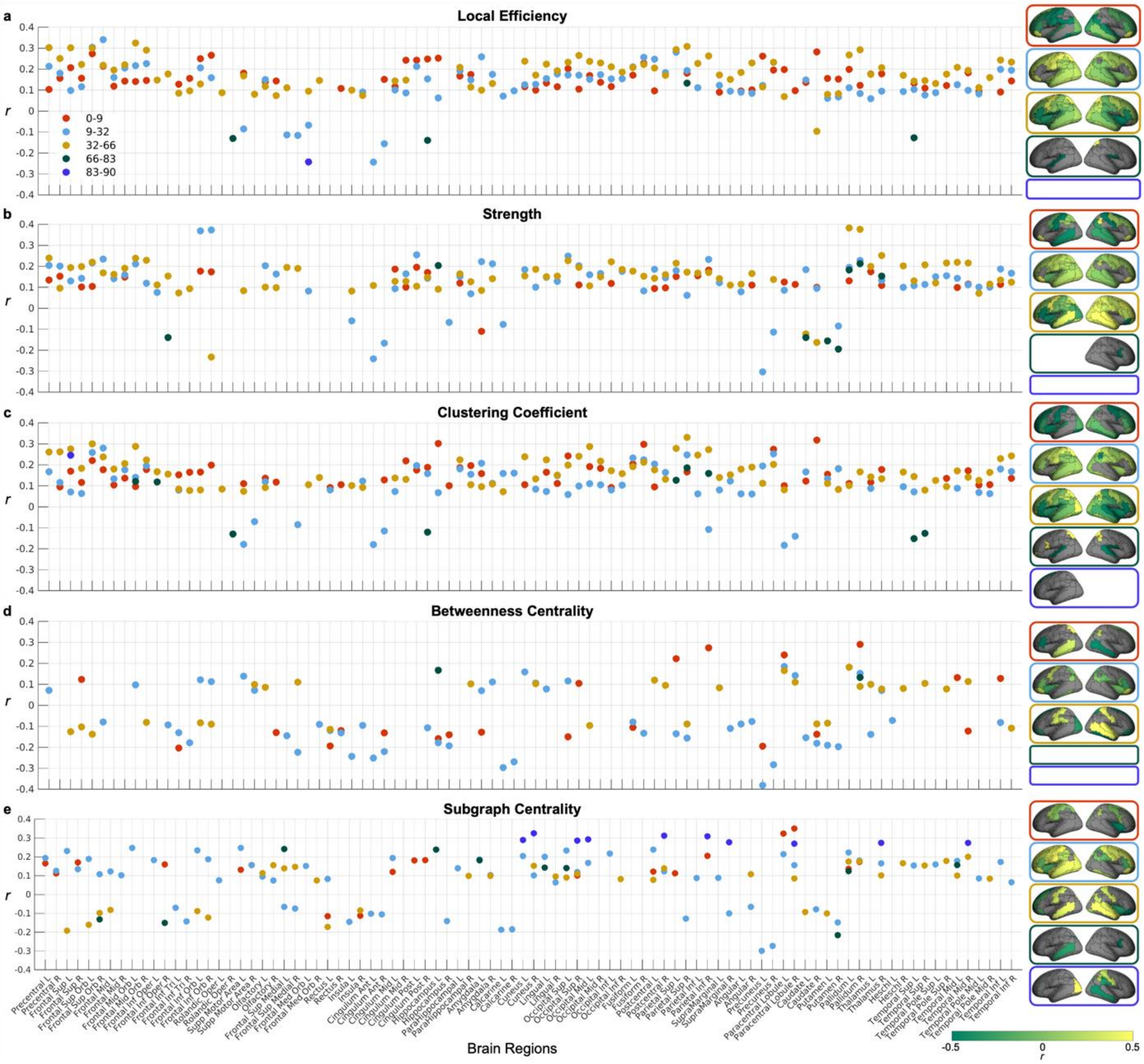

Supplementary Figure 6. Local correlations between organizational measures and age within each epoch. (a) Local efficiency was significant in 61 regions from 0-9 years old (epoch one), 70 regions from 9-32 years old (epoch two), 74 regions from 32-66 years old (epoch three), four regions from 66-83 years old (epoch four), and one region from 83-90 years old (epoch five). **(b)** Strength was significant in 31 regions in epoch one, 68 regions in epoch two, 71 regions in epoch three, eight regions in epoch four, and no regions in epoch five. **(c)** Clustering coefficient was significant in 55 regions in epoch one, 65 regions in epoch two, 71 regions in epoch three, nine regions in epoch four, and one region in epoch five. **(d)** Betweenness centrality was significant in 22 regions in epoch one, 50 regions in epoch two, 30 regions in epoch three, two regions in epoch four, and no regions in epoch five. **(e)** Subgraph centrality was significant in 19 regions in epoch one, 63 regions in epoch two, 35 regions in epoch three, 10 regions in epoch four, and 10 regions in epoch five. Values shown are the *r* values of all significant correlations after FDR correction. The surface plots show *r* values for each epoch (indicated by the color of the border). No surface plot indicates no significant correlations are present in the regions visible from the lateral orientations shown.

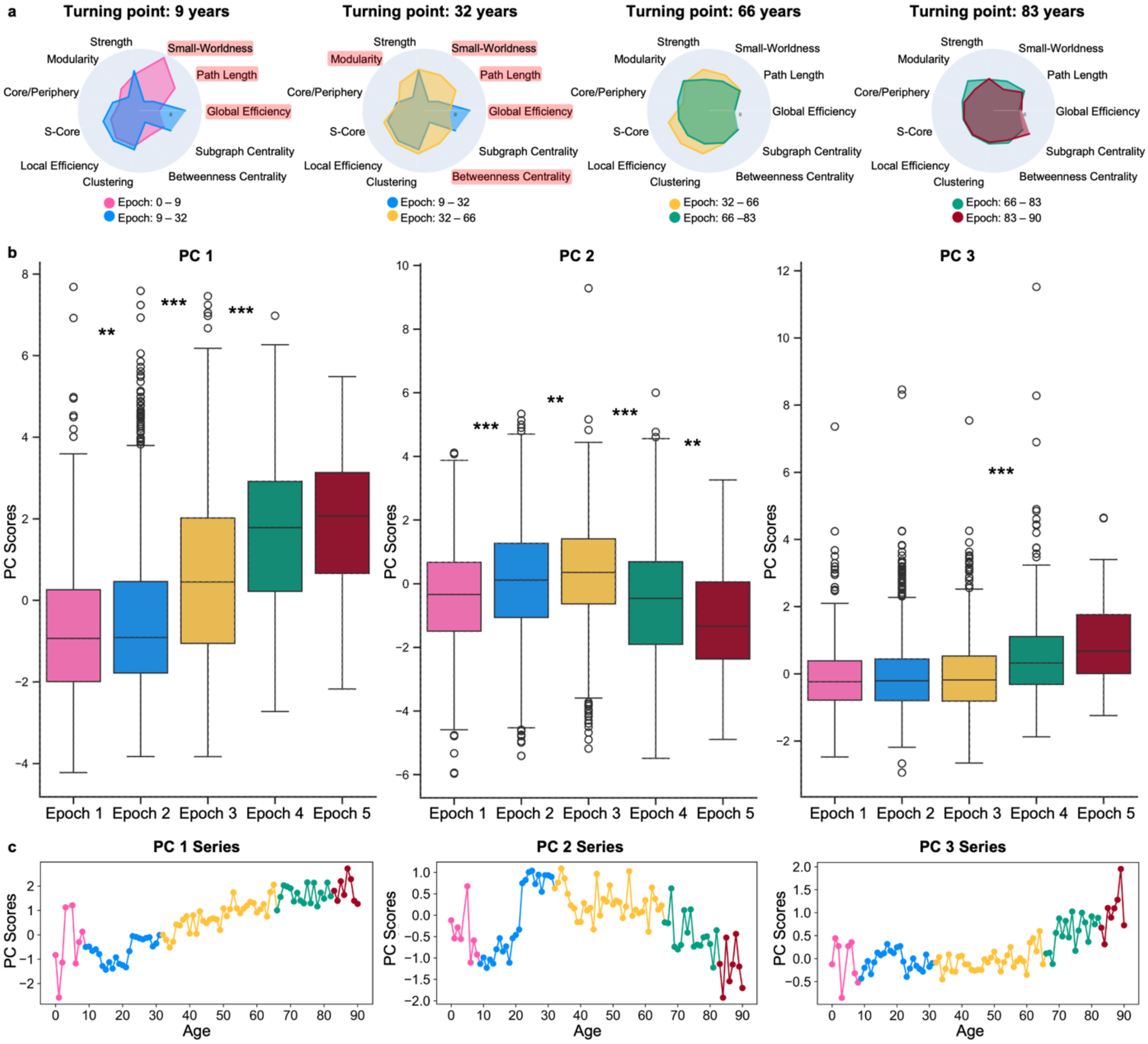

Supplementary Figure 7. Additional visualizations of epoch-based analyses. (a) Spider plots of Pearson *r* values for each graph theory measure in consecutive epochs highlight the epochs where the direction of significant age-topology relationship (indicated by red highlighted measures. **(b)** PCA scores per epoch across each PC (assessed via Welch's ANOVA). Across PC 1 scores, epochs one and two ($p = 0.002$), two and three ($p = 4.95 \times 10^{-13}$), and three and four ($p = 1.01 \times 10^{-14}$) are all significantly different. Similarly, for PC 2 scores, epochs one and two ($p = 2.15 \times 10^{-10}$), two and three ($p = 0.002$), and three and four ($p = 2.47 \times 10^{-13}$). Lastly, epochs three and four ($p = 1.82 \times 10^{-13}$) significantly differ across PC 3 scores, while epochs four and five only significantly differ in PC 2 scores ($p = 0.008$). **(c)** PC score series were created from average score for each age. These series were used in the Dynamic Time Warping analysis to explore the shape of trajectories. *** indicates $p < 0.001$, ** indicates $p < 0.01$, * indicates $p < 0.05$.

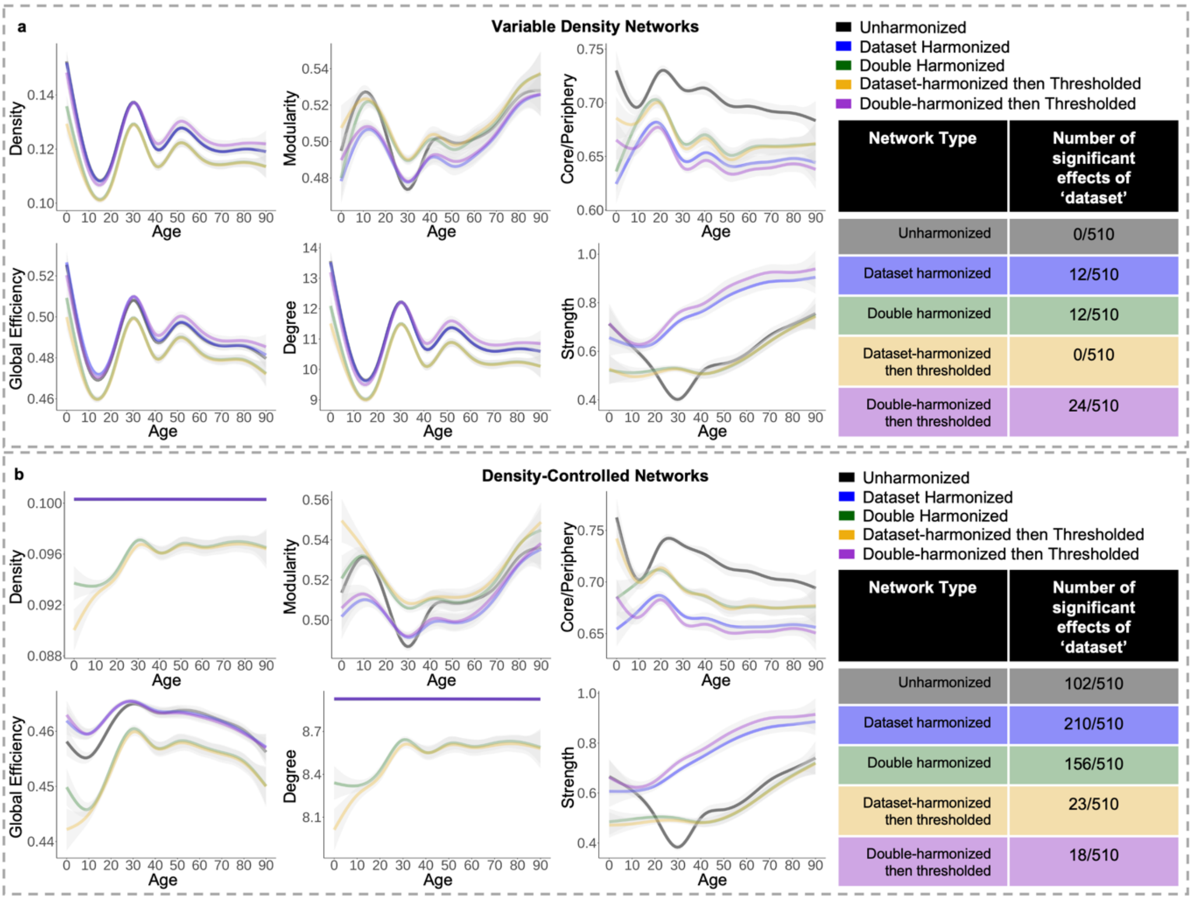

Network Type	Number of significant effects of 'dataset'
Unharmonized	0/510
Dataset harmonized	12/510
Double harmonized	12/510
Dataset-harmonized then thresholded	0/510
Double-harmonized then thresholded	24/510

Network Type	Number of significant effects of 'dataset'
Unharmonized	102/510
Dataset harmonized	210/510
Double harmonized	156/510
Dataset-harmonized then thresholded	23/510
Double-harmonized then thresholded	18/510

Supplementary Figure 8. Assessment of harmonization methods. (a) Variable density networks and **(b)** density-controlled networks with five different harmonization methods compared across density, modularity, core/periphery, global efficiency, average degree, and average strength across age. 'Double' harmonized refers to harmonizing across atlas and then dataset. The tables include the number of significant effects of dataset within age bins across all six measures after FDR correction.

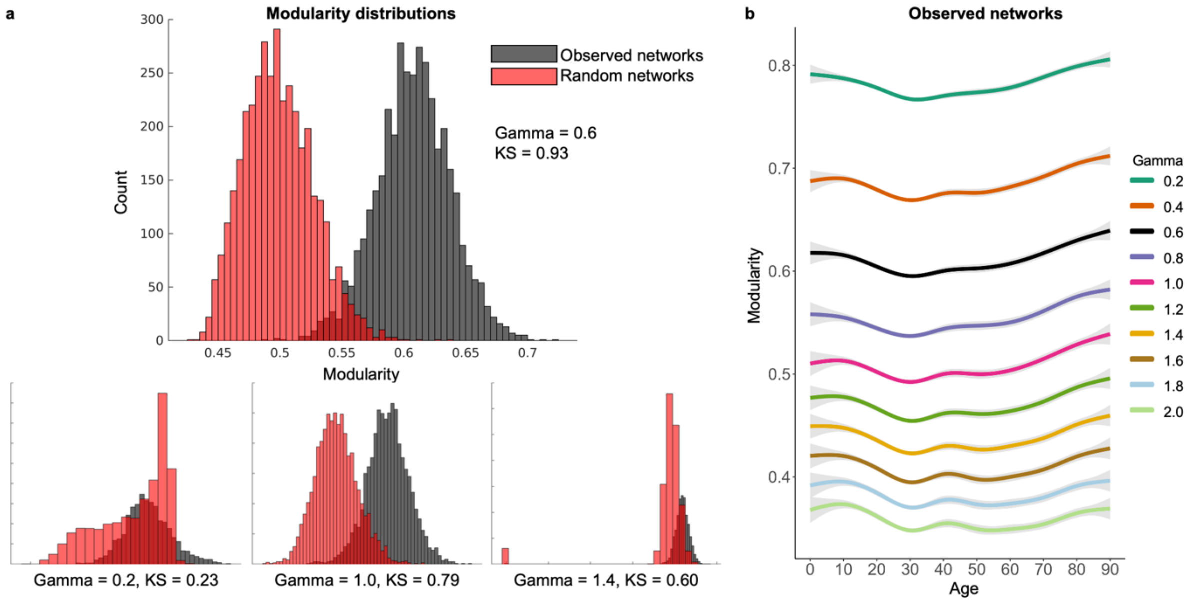

Supplementary Figure 9. Modularity at varying spatial resolutions. (a) The distributions of modularity at differing spatial resolutions (gamma) are compared between observed and randomized networks (with preserved density and degree distributions). KS is the Kolmogorov-Smirnov statistic, and all these distributions are significantly different ($p < 0.001$). **(b)** Generalized additive models of modularity across age at each level of gamma highlight that the location of inflection points is highly consistent across spatial resolution.

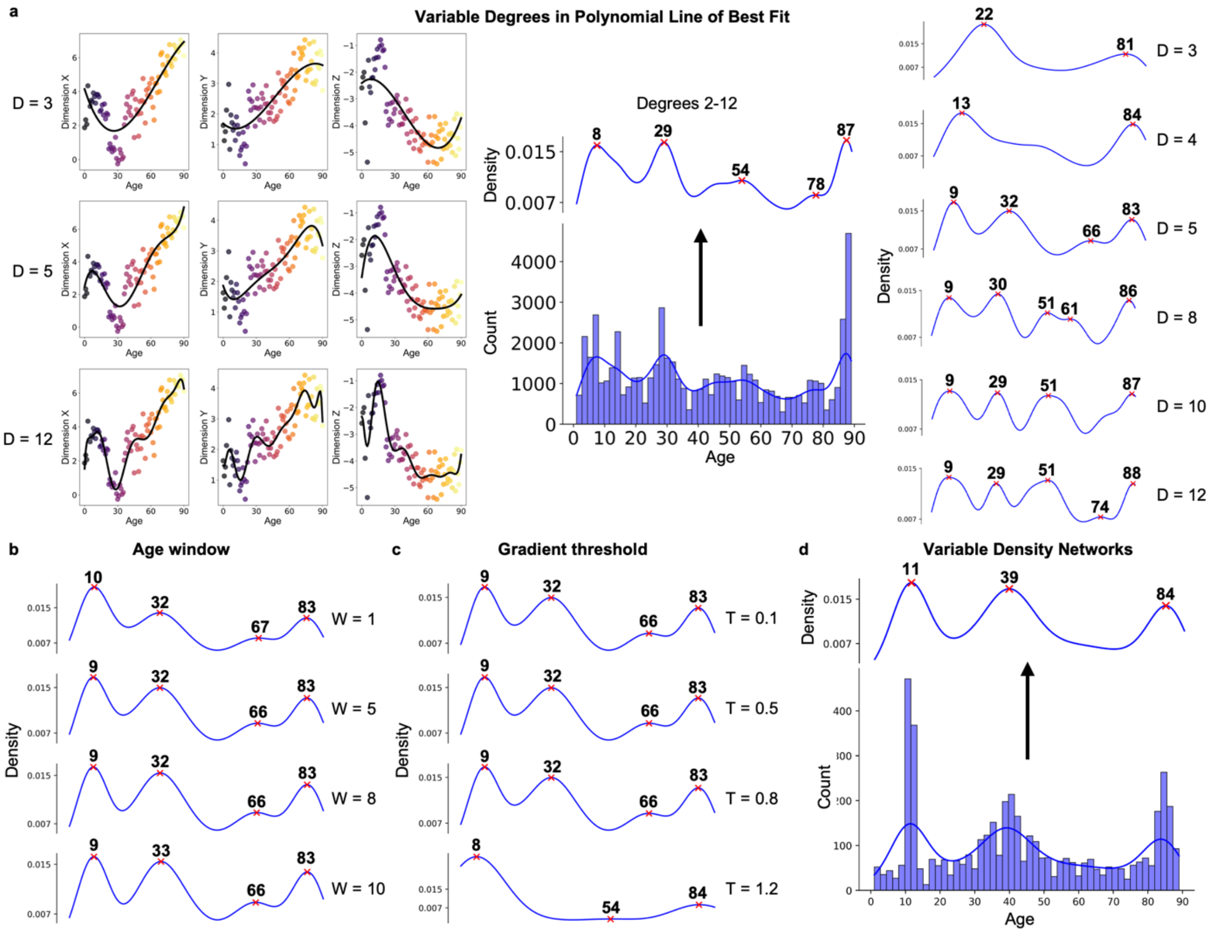

Supplementary Figure 10. Sensitivity analyses for turning points. (a) On the left is the manifold space with polynomial lines of best fit with degrees three, five, and 12. Visual inspection of these lines led to the decision to run the primary analysis with degree five. In the center, a histogram and density plot for all turning points identified with lines of best fit with degrees 2-12. Turning point density plots for lines of best fit with specific degrees on the right. These highlight that low degree lines (< 5) capture a teen to 20-year-old turning point, which disappears for higher degree lines – likely separating into the 8–9-year-old and 30–32-year-old turning points. High degree lines (> 8) additionally include two middle-to-late age turning points, falling between 50-74 years old, whereas a degree of 5 only has 66 years old as a turning point. **(a)** Multiple turning point density plots with various age windows demonstrate that from one to 10-year age windows, the four major turning points are highly consistent. **(c)** Multiple turning point density plots with various gradient thresholds highlight that the four turning points are stable across most thresholds. **(d)** Histogram and density plots for turning points identified in UMAP projection of variable density network topology. These sensitivity analyses suggest robust identification of turning points falling around 9, 32, and 83 years old.

Supplementary Table 5. Games-Howell statistics comparing PCA scores between epochs.

	Group A	Group B	Mean Diff.	SE	T-value	df	*p-value*	*Cohen's D*
**PC1**	Epoch 1	Epoch 2	-0.28	0.08	-3.66	1368.87	0.002	-0.16
	Epoch 1	Epoch 3	-1.40	0.09	-15.33	1667.87	6.44×10^{-13}	-0.74
	Epoch 1	Epoch 4	-2.45	0.11	-21.89	840.60	$<1.00 \times 10^{-323}$	-1.36
	Epoch 1	Epoch 5	-2.63	0.20	-13.33	115.12	3.62×10^{-14}	-1.50
	Epoch 2	Epoch 3	-1.12	0.08	-14.57	1754.62	4.95×10^{-13}	-0.60
	Epoch 2	Epoch 4	-2.17	0.10	-21.60	618.34	$<1.00 \times 10^{-323}$	-1.21
	Epoch 2	Epoch 5	-2.35	0.19	-12.30	101.32	3.55×10^{-14}	-1.33
	Epoch 3	Epoch 4	-1.05	0.11	-9.33	873.52	1.01×10^{-14}	-0.53
	Epoch 3	Epoch 5	-1.23	0.20	-6.21	115.57	8.58×10^{-08}	-0.61
	Epoch 4	Epoch 5	-0.18	0.20	-0.86	140.01	0.912	-0.09
**PC2**	Epoch 1	Epoch 2	-0.49	0.07	-6.75	1470.81	2.15×10^{-10}	-0.28
	Epoch 1	Epoch 3	-0.74	0.08	-9.18	1619.65	$<1.00 \times 10^{-323}$	-0.45
	Epoch 1	Epoch 4	0.09	0.11	0.83	774.79	0.920	0.05
	Epoch 1	Epoch 5	0.82	0.20	4.06	110.22	8.49×10^{-04}	0.50
	Epoch 2	Epoch 3	-0.25	0.07	-3.71	2018.37	0.002	-0.15
	Epoch 2	Epoch 4	0.58	0.10	5.69	609.27	1.96×10^{-07}	0.32
	Epoch 2	Epoch 5	1.30	0.20	6.64	100.63	1.63×10^{-08}	0.74
	Epoch 3	Epoch 4	0.83	0.11	7.71	740.27	2.47×10^{-13}	0.47
	Epoch 3	Epoch 5	1.56	0.20	7.80	107.51	4.28×10^{-11}	0.91
	Epoch 4	Epoch 5	0.72	0.21	3.40	138.59	0.008	0.38
**PC3**	Epoch 1	Epoch 2	-0.06	0.04	-1.30	1449.43	0.692	-0.06
	Epoch 1	Epoch 3	-0.08	0.05	-1.66	1647.78	0.462	-0.08
	Epoch 1	Epoch 4	-0.67	0.08	-8.84	678.36	2.73×10^{-13}	-0.59
	Epoch 1	Epoch 5	-1.05	0.13	-8.03	107.42	1.31×10^{-11}	-1.05
	Epoch 2	Epoch 3	-0.03	0.04	-0.62	1914.28	0.972	-0.02
	Epoch 2	Epoch 4	-0.61	0.07	-8.63	550.34	5.30×10^{-13}	-0.55
	Epoch 2	Epoch 5	-0.99	0.13	-7.76	99.10	7.76×10^{-11}	-0.94
	Epoch 3	Epoch 4	-0.59	0.08	-7.81	669.64	1.82×10^{-13}	-0.50
	Epoch 3	Epoch 5	-0.96	0.13	-7.42	106.44	2.98×10^{-10}	-0.89
	Epoch 4	Epoch 5	-0.38	0.14	-2.66	148.10	0.065	-0.28

Note: 'Mean Diff.' is the difference between mean Epoch PCA scores. 'SE' is the standard error, and 'df' is the degrees of freedom. All values are uncorrected.
