# Supplementary material for: Topological turning points across the human lifespan
Source: Nat Commun. 2025 Nov 25;16:10055. doi: 10.1038/s41467-025-65974-8 (PMC12647875; doi:10.1038/s41467-025-65974-8)
Supplement: Supplementary file 2 — Reporting Summary [file 41467_2025_65974_MOESM2_ESM.pdf]

Corresponding author(s): Alexa Mousley

Last updated by author(s): October 3, 2025

Reporting Summary

Nature Portfolio wishes to improve the reproducibility of the work that we publish. This form provides structure for consistency and transparency in reporting. For further information on Nature Portfolio policies, see our Editorial Policies and the Editorial Policy Checklist.

Statistics

For all statistical analyses, confirm that the following items are present in the figure legend, table legend, main text, or Methods section.

n/a	Confirmed	
☐	☒	The exact sample size (*n*) for each experimental group/condition, given as a discrete number and unit of measurement
☐	☒	A statement on whether measurements were taken from distinct samples or whether the same sample was measured repeatedly
☐	☒	The statistical test(s) used AND whether they are one- or two-sided<br>*Only common tests should be described solely by name; describe more complex techniques in the Methods section.*
☐	☒	A description of all covariates tested
☐	☒	A description of any assumptions or corrections, such as tests of normality and adjustment for multiple comparisons
☐	☒	A full description of the statistical parameters including central tendency (e.g. means) or other basic estimates (e.g. regression coefficient) AND variation (e.g. standard deviation) or associated estimates of uncertainty (e.g. confidence intervals)
☐	☒	For null hypothesis testing, the test statistic (e.g. *F*, *t*, *r*) with confidence intervals, effect sizes, degrees of freedom and *P* value noted<br>*Give P values as exact values whenever suitable.*
☒	☐	For Bayesian analysis, information on the choice of priors and Markov chain Monte Carlo settings
☐	☒	For hierarchical and complex designs, identification of the appropriate level for tests and full reporting of outcomes
☐	☒	Estimates of effect sizes (e.g. Cohen's *d*, Pearson's *r*), indicating how they were calculated

Our web collection on statistics for biologists contains articles on many of the points above.

Software and code

Policy information about availability of computer code

Data collection	No data was collected by the authors. CALM data are available at https://portal.camide.cam.ac.uk/overview/1158. Cam-CAN data are available at https://camcan-archive.mrc-cbu.cam.ac.uk/dataaccess/. BCP, dHCP, HCPd, HCPya, and HCPa data are available at https://nda.nih.gov/.
Data analysis	All analysis scripts have been published on GitHub: https://github.com/alexamousley/lifespan_topological_turning_points.<br>Software: DSI Studio "Chen" release<br>Code versions are: MATLAB 2020b; Python 3.7.3; RStudio 4.1.2

For manuscripts utilizing custom algorithms or software that are central to the research but not yet described in published literature, software must be made available to editors and reviewers. We strongly encourage code deposition in a community repository (e.g. GitHub). See the Nature Portfolio guidelines for submitting code & software for further information.

Data

Policy information about availability of data

All manuscripts must include a data availability statement. This statement should provide the following information, where applicable:

- Accession codes, unique identifiers, or web links for publicly available datasets
- A description of any restrictions on data availability
- For clinical datasets or third party data, please ensure that the statement adheres to our policy

The derived data generated in this study are available at https://osf.io/7p4y3/. CALM data are available at https://portal.camide.cam.ac.uk/overview/1158. BCP data

are available at https://nda.nih.gov/. The semi-processed data from dHCP, HCPd, HCPya, HCPa, and Cam-CAN used in this publication are available at https://brain.labsolver.org/.

Research involving human participants, their data, or biological material

Policy information about studies with human participants or human data. See also policy information about sex, gender (identity/presentation), and sexual orientation and race, ethnicity and racism.

Reporting on sex and gender	Sex data (as a biological attribute) was not directly relevant to the research questions of interested in this is paper but was used as a covariate in Generalized Additive Models. No gender data was accessed.
Reporting on race, ethnicity, or other socially relevant groupings	No race, ethnicity or social data was accessed.
Population characteristics	The population characteristic used was age. Age was examined rounded to the closest year by converting age reported in weeks (dHCP) or months (e.g., HCPd) to the nearest year.
Recruitment	There was no recruitment for this project.
Ethics oversight	Given that we did not collect data, there were no ethics oversight for this project directly. Ethics for individual datasets can be found on their sites.

Note that full information on the approval of the study protocol must also be provided in the manuscript.

Field-specific reporting

Please select the one below that is the best fit for your research. If you are not sure, read the appropriate sections before making your selection.

- [x] Life sciences
- [ ] Behavioural & social sciences
- [ ] Ecological, evolutionary & environmental sciences

For a reference copy of the document with all sections, see nature.com/documents/nr-reporting-summary-flat.pdf

Life sciences study design

All studies must disclose on these points even when the disclosure is negative.

Sample size	The original sample was 4,216 across nine datasets as this was the total number of scans we had access to with matched age and sex data.
Data exclusions	The analysis sample size was 3,802 participants across nine datasets. The original sample was 4,216, from which we removed 87 repeat or longitudinal scans from BCP. We also excluded 313 participants from the CALM dataset for being identified as individuals with challenges in attention, learning or memory. We also removed 14 outliers, identified by having network density above or below three standard deviations for their age. 12 participants from HCPa were excluded as their age was 100+, but there were no participants between 90-100.
Replication	Measures have been taken the maximize transparency for the sake of future reproducibility. All code is available at https://github.com/alexamousley/lifespan_topological_turning_points and derived data at https://osf.io/7p4y3/.
Randomization	No randomization was required as this was a study of normative development.
Blinding	No blinding was required due to it being a normative modeling study with no 'atypical' or 'control' groups.

Reporting for specific materials, systems and methods

We require information from authors about some types of materials, experimental systems and methods used in many studies. Here, indicate whether each material, system or method listed is relevant to your study. If you are not sure if a list item applies to your research, read the appropriate section before selecting a response.

Materials & experimental systems

n/a	Involved in the study	
x		Antibodies
x		Eukaryotic cell lines
x		Palaeontology and archaeology
x		Animals and other organisms
x		Clinical data
x		Dual use research of concern
x		Plants

Methods

n/a	Involved in the study	
x		ChIP-seq
x		Flow cytometry
	x	MRI-based neuroimaging

Plants

Seed stocks	*Report on the source of all seed stocks or other plant material used. If applicable, state the seed stock centre and catalogue number. If plant specimens were collected from the field, describe the collection location, date and sampling procedures.*
Novel plant genotypes	*Describe the methods by which all novel plant genotypes were produced. This includes those generated by transgenic approaches, gene editing, chemical/radiation-based mutagenesis and hybridization. For transgenic lines, describe the transformation method, the number of independent lines analyzed and the generation upon which experiments were performed. For gene-edited lines, describe the editor used, the endogenous sequence targeted for editing, the targeting guide RNA sequence (if applicable) and how the editor was applied.*
Authentication	*Describe any authentication procedures for each seed stock used or novel genotype generated. Describe any experiments used to assess the effect of a mutation and, where applicable, how potential secondary effects (e.g. second site T-DNA insertions, mosiacism, off-target gene editing) were examined.*

Magnetic resonance imaging

Experimental design

Design type	Diffusion MRI
Design specifications	Each of the nine datasets had different MRI design specifications which are available in Extended Data Table 1.
Behavioral performance measures	No behavioral tasks were accessed.

Acquisition

Imaging type(s)	Diffusion
Field strength	Variable per study
Sequence & imaging parameters	Imaging sequence and parameters varied across the nine studies.
Area of acquisition	Whole-brain scans

Diffusion MRI ☒ Used ☐ Not used

Parameters: dMRI parameters differed per study. The information is reported in Extended Data Table 1

Preprocessing

Preprocessing software	Data was preprocessed differently depending on the dataset. Some were preprocessed by Dr Yeh and made available via the Fiber Data Hub - preprocessing information for these studies is available at https://brain.labsolver.org/. Other studies were processed using QSIprep (https://qsiprep.readthedocs.io/en/latest/)
Normalization	Data was preprocessed differently depending on the dataset. Some were preprocessed by Dr Yeh and made available via the Fiber Data Hub - preprocessing information for these studies is available at https://brain.labsolver.org/. Other studies were processed using QSIprep (https://qsiprep.readthedocs.io/en/latest/)
Normalization template	Data was preprocessed differently depending on the dataset. Some were preprocessed by Dr Yeh and made available via the Fiber Data Hub - preprocessing information for these studies is available at https://brain.labsolver.org/. Other studies were processed using QSIprep (https://qsiprep.readthedocs.io/en/latest/)
Noise and artifact removal	Data was preprocessed differently depending on the dataset. Some were preprocessed by Dr Yeh and made available via the Fiber Data Hub - preprocessing information for these studies is available at https://brain.labsolver.org/. Other studies were processed using QSIprep (https://qsiprep.readthedocs.io/en/latest/)
Volume censoring	No volume censoring was performed by the authors.

Statistical modeling & inference

Model type and settings	Generalized additive models (GAMs) with penalized splines were performed with the mgcv package in R v4.1.2. Models were fit with the restricted maximum likelihood (REML) method. Cubic regression splines were used as the basis function. The curvature of the model was assessed by fitting the model with cubic shrinkage splines. Sex, atlas and dataset were controlled for in GAMs when looking at changes across age. We also used regularized Least Absolute Shrinkage and Selection Operator (LASSO) to look at how graph theory metrics predict age within lifespan epochs. Additionally, we performed principal components analysis (PCA), uniform manifold approximation and projection (UMAP), and dynamic time warping (DTW) to explore changes in topology across age.
Effect(s) tested	We explored the effects of age (years) on graph theory metrics.

Specify type of analysis: ☒ Whole brain ☐ ROI-based ☐ Both

Statistic type for inference

(See Eklund et al. 2016)

Local and global graph theory metrics calculated with 4 version of the AAL90 atlas (Shi, F. et al. Infant brain atlases from neonates to 1- and 2-year-olds. PLoS One 6, e18746 (2011)).

Correction

No corrections were applied.

Models & analysis

n/a	Involved in the study
☒	☐ Functional and/or effective connectivity
☐	☒ Graph analysis
☐	☒ Multivariate modeling or predictive analysis

Graph analysis

We used participant-level normalized weighted graphs.

Multivariate modeling and predictive analysis

We explored multiple graph theory metrics: density, strength, global efficiency, maximum modularity, small-worldness, characteristic path length, local efficiency, s-core, k-core, clustering coefficient, core/periphery, betweenness centrality, and subgraph centrality. We used Generalized Additive Models to predict these metrics from age. We also used regularized Least Absolute Shrinkage and Selection Operator to predict age from graph theory metrics within age ranges (epochs).
